# Risk of myeloid neoplasms after radiotherapy among older women with localized breast cancer: A population-based study

**DOI:** 10.1371/journal.pone.0184747

**Published:** 2017-09-13

**Authors:** Amer M. Zeidan, Jessica B. Long, Rong Wang, Xin Hu, James B. Yu, Scott F. Huntington, Gregory A. Abel, Sarah S. Mougalian, Nikolai A. Podoltsev, Steven D. Gore, Cary P. Gross, Xiaomei Ma, Amy J. Davidoff

**Affiliations:** 1 Department of Internal Medicine, School of Medicine, Yale University, New Haven, Connecticut, United States of America; 2 Cancer Outcomes, Public Policy, and Effectiveness Research (COPPER) Center, Yale University, New Haven, Connecticut, United States of America; 3 Department of Chronic Disease Epidemiology, School of Public Health, Yale University, New Haven, Connecticut, United States of America; 4 Department of Therapeutic Radiology, Yale University, New Haven, Connecticut, United States of America; 5 Division of Population Sciences, Department of Medical Oncology, Dana-Farber Cancer Institute, Boston, Massachusetts, United States of America; 6 Department of Health Policy and Management, School of Public Health, Yale University, New Haven, Connecticut, United States of America; Cardiff University, UNITED KINGDOM

## Abstract

**Background:**

There are inconsistent and limited data regarding the risk of myeloid neoplasms (MN) among breast cancer survivors who received radiotherapy (RT) in the absence of chemotherapy. Concern about subsequent MN might influence the decision to use adjuvant RT for women with localized disease. As patients with therapy-related MN have generally poor outcomes, the presumption of subsequent MN being therapy-related could affect treatment recommendations.

**Methods:**

We used the Surveillance, Epidemiology, and End Results (SEER)–Medicare linked database to study older women with in-situ or stage 1–3 breast cancer diagnosed 2001–2009 who received surgery. Chemotherapy and RT were ascertained using Medicare claims, and new MN diagnoses were captured using both SEER registry and Medicare claims. We excluded women who received chemotherapy for initial treatment, and censored at receipt of subsequent chemotherapy. Competing-risk survival analysis was used to assess the association between RT and risk of subsequent MN adjusting for relevant characteristics.

**Results:**

Median follow-up for 60,426 eligible patients was 68 months (interquartile range, 46 to 92 months), with 47.6% receiving RT. In total, 316 patients (0.52%) were diagnosed with MN; the cumulative incidence per 10,000 person-years was 10.6 vs 9.0 among RT-treated vs non-RT-treated women, respectively (p = .004); the increased risk of subsequent MN persisted in the adjusted analysis (hazard ratio = 1.36, 95% confidence interval: 1.03–1.80). The results were consistent in multiple sensitivity analyses.

**Conclusions:**

Our data suggest that RT is associated with a significant risk of subsequent MN among older breast cancer survivors, though the absolute risk increase is very small. These findings suggest the benefits of RT outweigh the risks of development of subsequent MN.

## Introduction

Breast cancer is the most frequently diagnosed malignancy among women in the United States, with 41% of patients diagnosed at age 65 years or older[1]. Adjuvant radiotherapy (RT), neoadjuvant and adjuvant chemotherapy, and/or hormonal therapy are the commonly used treatments for early stage (stages 1–3) disease in addition to surgical intervention for the primary tumor. One of the longer-term complications of chemotherapy is the development of secondary malignancies including myelodysplastic syndromes (MDS), acute myeloid leukemia (AML), and therapy-related MDS/myeloproliferative neoplasms overlap (t-MDS/MPN), which are grouped together as “therapy-related myeloid neoplasms” (t-MN)[2]. The occurrence of t-MN is a well-recognized complication among breast cancer survivors who undergo adjuvant chemotherapy or combined chemotherapy and RT[3–7]. Moreover, given that breast cancer is common and associated with high rates of long-term survivorship, a significant proportion of all t-MN cases occur in breast cancer survivors[7].

Concern about subsequent t-MN development is part of the risk-benefit calculation in decisions about chemotherapy use, especially in older patients, but it is unclear whether RT without chemotherapy increases t-MN. Radiotherapy has been hypothesized to promote MN development by causing reactive oxygen species formation which may lead to double-strand DNA breaks and genomic instability. However, breast RT largely spares hematopoietic tissue, suggesting that any effects would be small. There is conflicting empirical evidence regarding whether adjuvant RT increases the MN risk in the absence of chemotherapy[1–9]. Indeed, observing differences in the risk for rare malignancies that occur with a medium to long term latency after exposure (at least 5 years) is difficult. Prior studies have been limited by short follow-up; relatively small samples and/or highly selected patients (e.g., clinical trial participants); incomplete capture of additional chemotherapy exposure; use of controls from the general population not accounting for increased risk of second malignancy among individuals with a prior cancer diagnosis; or were completed before dissemination of newer RT modalities associated with reduced radiation exposure[1, 3, 5–8].

We conducted a large population-based study focusing exclusively on older women who underwent surgical resection for localized breast cancer. We compared women who received RT with those who did not, excluding women who received chemotherapy as part of their initial treatment. We aimed to ensure a long follow-up period, and used two distinct algorithms to capture key exposures and outcomes so that we could perform extensive sensitivity analyses. Given its localized application and the fact that radiation for breast cancer largely spares hematopoietic tissue and the results of recent studies,[7,8] we hypothesized that among women who did not receive chemotherapy, receiving RT would not be associated with a higher incidence of subsequent MN.

## Methods

### Data source and study population

The Surveillance, Epidemiology, and End Results (SEER) program provides data regarding cancer diagnoses and selected demographics from tumor registries across the United States (US). The SEER data are linked at the individual level to Medicare data on enrollment, demographic and socioeconomic characteristics, and claims[10, 11]. We used the linked SEER-Medicare database to conduct a retrospective cohort study of older female breast cancer patients who were diagnosed at age 67 to 94 years with in-situ or stage 1–3 breast cancer between 1/1/2001 and 12/31/2009. We required a primary breast cancer diagnosis by December 2009 to ensure every subject had a minimum potential follow-up for 3 years. Eligible patients included women who: 1) were enrolled in Medicare Parts A and B and fee-for-service continuously from 24 months before diagnosis through death or end of study; 2) underwent surgery for breast cancer within 9 months of diagnosis; 3) not diagnosed with other neoplasms (including MN) prior to breast cancer; 4) did not receive RT after initial treatment window (which could indicate recurrence or diagnoses of a subsequent cancer); and 5) did not receive chemotherapy as part of their initial breast cancer therapy (from breast diagnosis through 9 months after the primary breast cancer surgery).

### Variable construction

We used a combination of SEER and claims data using the presence of specific International Classification of Diseases version 9, clinical modification (ICD-9-CM) diagnosis codes and Healthcare Common Procedural Coding System (HCPCS) codes from Medicare claims to define our primary outcome, exposure, and other covariates, with alternative versions for outcome and exposure based on SEER data alone. Our primary outcome measure, diagnosis of MN, was identified based on a recorded diagnosis of MDS, AML or other MN in SEER records (International Classification of Disease for Oncology, 3^rd^ edition [ICD-O-3][12]) or Medicare claims (S1 Table). The category of other MN included chronic myeloid leukemia, chronic myelomonocytic leukemia, and overlap MPN/MDS. Chronic myelomonocytic leukemia and overlap MPN/MDS were classified within MDS prior to 2008 under WHO classification, so we included them to ensure consistency in our outcome over time. We also included chronic myeloid leukemia as it is a myeloid malignancy previously observed to be associated with RT receipt[13].

To identify MN from claims, we applied the following algorithm: 1) MN diagnosis code on an inpatient claim or two outpatient claims >30 (but < 365) days apart; AND 2) a claim for bone marrow aspirate or biopsy within 60 days before or after the initial MN diagnosis; AND 3) at least one claim with MN diagnosis after aspirate or biopsy claim. If a woman was identified with MN by both SEER and claims, then we used the earlier date[12]. Our alternative measure relied solely on SEER diagnoses. Our primary measure of RT receipt required any claims with a treatment delivery code for brachytherapy or ≥ 4 claims with treatment delivery codes for external beam RT, initiated within nine months of diagnosis[14]. Our alternative measure used only SEER indication of RT as initial treatment modality.

We utilized the following variables from SEER data: age, marital status, year of breast cancer diagnosis, breast cancer characteristics (stage, grade, size, lymph node involvement, hormone receptor status, laterality), and median household income and education at the census tract level (with zip code level measures assigned if census tract measures were missing). Race is provided by SEER separately from Hispanic ethnicity. We categorized race as white, black, or other, with people of Hispanic ethnicity categorized by their listed racial category[15]. We determined whether each woman resided in a metropolitan county using the 2003 Rural-Urban Continuum Code which is supplied by SEER. We assessed additional variables using Medicare claims including comorbid conditions[16] and disability status[17]. We identified individuals with anemia pre-diagnosis using the same algorithm as for medical comorbidities in the 24 to 3 months prior to breast cancer diagnosis using ICD-9-CM diagnosis codes with the first 3 digits 280–285. We also used claims to assess receipt and type of breast surgery and radiation (initial and late). The Yale Human Investigation Committee determined that our study did not directly involve human subjects.

### Statistical analysis

Patients were followed from breast cancer diagnosis through the earliest diagnosis of a second malignancy, death or end of study (December 31, 2010 for those diagnosed 2001–2003 and December 31, 2012 for those diagnosed 2004–2009). Frequency distribution of demographic and socioeconomic characteristics across treatment groups were compared using Chi-square tests.

We summarized number of events and years of follow-up for each treatment group (overall, RT and no-RT groups) to calculate incidence rate and used bootstrapping to generate 95% confidence intervals (CIs). For all time-to-event analyses, we have used the Fine-Gray sub-distribution method in our incidence rate and time-to-event models to account for competing risks of death and developing a second malignancy other than MN.[18] Because chemotherapy conveys a separate risk of MN, we censored patients at the time they received late chemotherapy (>9 months after breast surgery) except when chemotherapy was administered within 30 days of a SEER or algorithm-based MN diagnosis. We censored at the end of follow-up. We assessed the proportional hazard assumption in the primary models using interaction of time with each variable and found no significant deviation from the assumption. Sensitivity analyses used alternative (SEER only) measures of exposure and outcome. We also conducted sensitivity analyses in subgroups based on breast cancer stage and node involvement, to account for likely higher doses and/or or larger RT fields. We included variables associated with RT receipt or outcome in our models. All analyses were two-sided and conducted using SAS Version 9.4 (SAS Inc. Cary, North Carolina), with an alpha of 0.05.

## Results

### Study population

**Fig 1:**
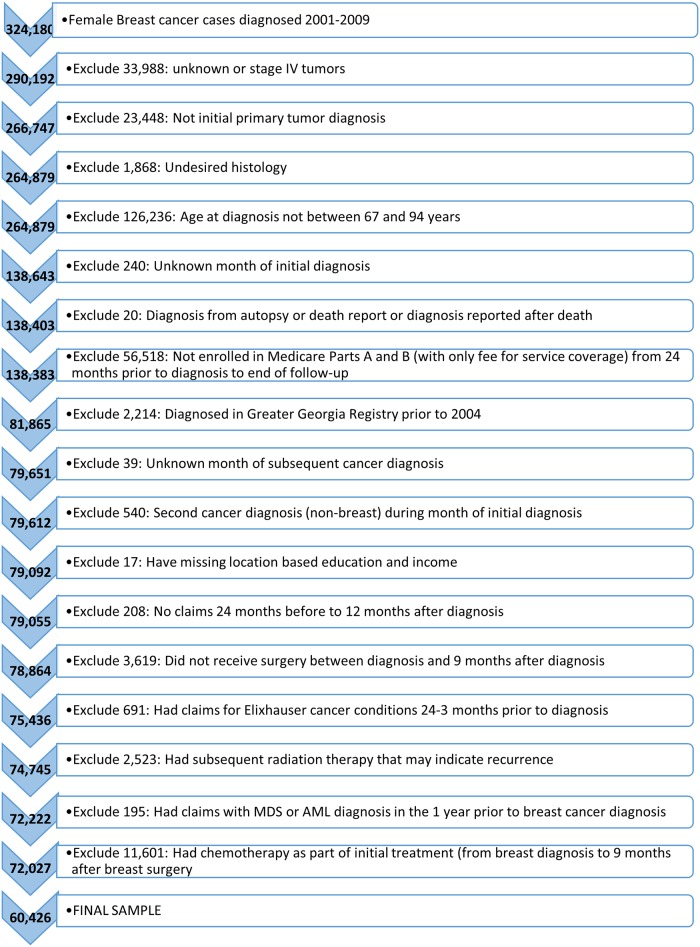
Creation of study sample.

A total of 60,426 patients met eligibility criteria and were included in the study [Fig 1]. Median follow-up for all individuals was 68 months (interquartile range [IQR], 46 to 92 months) for a total of 320,928 person-years. Almost half (28,759 patients, 47.6%) received RT as initial treatment after surgical resection of primary tumor. Median follow-up for women who received RT was 74 (IQR, 52–94) months, compared to 62 (IQR, 41–89) months in women who did not receive RT (p <.001). Women who received RT were younger, more likely to be married, have fewer comorbidities, more likely to have stage I disease with smaller tumors, and more likely to receive breast conserving surgery (BCS) (all p <.001, Table 1).

**Table 1:** Characteristics of study sample, stratified by receipt of radiation therapy.

	RT		No RT		Chi-square
	N	%	N	%	P-value
Total	28,759	47.6%	31,667	52.4%	
Age					
67–69	4,923	17.1%	3,467	10.9%	<.001
70–74	8,576	29.8%	6,499	20.5%	
75–79	8,119	28.2%	7,743	24.5%	
80–84	5,248	18.2%	7,406	23.4%	
85+	1,893	6.6%	6,552	20.7%	
Race					
White	26,229	91.2%	28,360	89.6%	<.001
Black	1,427	5.0%	2,078	6.6%	
Other	1,103	3.8%	1,229	3.9%	
Marital Status					
Married	13,931	48.4%	11,531	36.4%	<.001
Unmarried	13,914	48.4%	18,901	59.7%	
Unknown	914	3.2%	1,235	3.9%	
Median household income					
< $33,000	4,656	16.2%	7,046	22.3%	<.001
$33,000–40,000	3,883	13.5%	5,168	16.3%	
$40,000–50,000	5,719	19.9%	6,610	20.9%	
$50,000–63,000	6,133	21.3%	5,867	18.5%	
≥ $63,000	8,368	29.1%	6,976	22.0%	
Education (% adults with High School diploma or less)					
<30%	8,883	30.9%	7,733	24.4%	<.001
30 to < 40%	5,146	17.9%	5,036	15.9%	
40 to <50%	5,014	17.4%	5,393	17.0%	
50 to < 60%	4,558	15.8%	5,708	18.0%	
≥ 60%	5,158	17.9%	7,797	24.6%	
Residence in Metropolitan Area	24,986	86.9%	25,614	80.9%	<.001
Elixhauser Comorbidity					
None	14,422	50.1%	13,203	41.7%	<.001
1 to 2	11,175	38.9%	12,592	39.8%	
3 or more	3,162	11.0%	5,872	18.5%	
Predicted Disability	1,008	3.5%	3,582	11.3%	<.001
Medicaid Dual Enrollment	10,509	36.5%	13,709	43.3%	<.001
Pre-diagnosis anemia	3,969	13.8%	5,673	17.9%	<.001
Stage					
In situ	5,307	18.5%	7,177	22.7%	<.001
I	17,008	59.1%	13,997	44.2%	
II	5,463	19.0%	8,874	28.0%	
III	981	3.4%	1,619	5.1%	
Grade					
Well differentiated	7,788	27.1%	7,167	22.6%	<.001
Moderately differentiated	12,463	43.3%	12,738	40.2%	
Poorly differentiated	5,516	19.2%	7,088	22.4%	
Undifferentiated	740	2.6%	935	3.0%	
Unknown	2,252	7.8%	3,739	11.8%	
Tumor size					
<2.0 cm	14,404	50.1%	12,065	38.1%	<.001
2.0–5.0 cm	3,950	13.7%	6,387	20.2%	
>5.0 cm	480	1.7%	894	2.8%	
Missing	9,925	34.5%	12,321	38.9%	
Lymph node involvement	3,447	12.0%	4,597	14.5%	<.001
Laterality					
Right	14,311	49.8%	15,434	48.7%	.016
Left	*14*,*437*	*50*.*2%*	*16*,*222*	51.2%	
Missing*	*11*	*0*.*0%*	*11*	0.0%	
Hormone receptor Status					
ER—and PR–	1,692	5.9%	2,216	7.0%	<.001
ER+ or PR+	16,740	58.2%	15,628	49.4%	
Missing	10,327	35.9%	13,823	43.7%	
Surgery Type					
Mastectomy	2,069	7.2%	20,281	64.0%	<.001
Breast Conserving Surgery	26,690	92.8%	11,386	36.0%	
Late Chemotherapy	1,962	6.8%	2,294	7.2%	.043
Year of Diagnosis					
2001	2,879	10.0%	3,782	11.9%	<.001
2002	2,807	9.8%	3,603	11.4%	
2003	3,106	10.8%	3,174	10.0%	
2004	3,189	11.1%	3,564	11.3%	
2005	3,150	11.0%	3,487	11.0%	
2006	3,379	11.7%	3,439	10.9%	
2007	3,462	12.0%	3,376	10.7%	
2008	3,503	12.2%	3,438	10.9%	
2009	3,284	11.4%	3,804	12.0%	

Source: SEER-Medicare. Abbreviations: RT: Radiation Therapy, ER: estrogen receptor, PR: progesterone receptor.

*N<11 edited to meet data use requirements.

### Diagnosis of MN and relationship to receipt of RT

During follow-up, a total of 316 patients (0.52%) were diagnosed with MN, with 176 cases occurring in individuals who received RT, while the remaining 140 cases were diagnosed among those who did not receive RT. This yielded a cumulative MN incidence of 10.6 (95% CI: 9.1–12.2) cases per 10,000 person-years in women who received RT compared to 9.0 (95% CI: 7.6–10.5 cases per 10,000 person-years in women who did not receive RT (p = .004). Most of these diagnoses were MDS (8.2 (95% CI: 6.9–9.6) in RT-receiving patients versus 6.3 (95% CI: 5.0–7.6) in RT-non-receiving patients per 10,000 person-years, p = .001).

**Fig 2:**
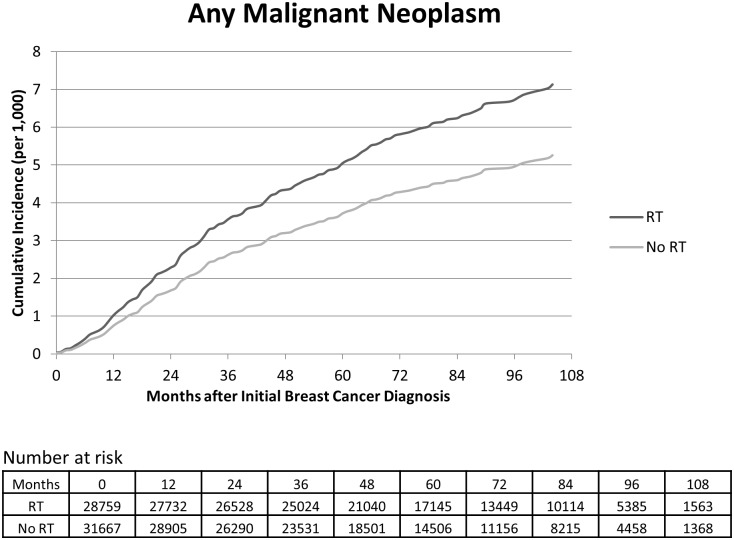
Adjusted cumulative incidence of myeloid neoplasm (MN) for localized breast cancer among older women stratified by receipt of radiation therapy. Source: SEER-Medicare. Source: SEER-Medicare. Abbreviations: RT: Radiation Therapy, BCS: Breast Conserving Surgery, MDS: myelodysplastic syndromes, AML: acute myeloid leukemia. Model adjusted for age, race, marital status, income, education, metropolitan status, comorbidity, disability, anemia, stage, grade, tumor size, node involvement, tumor laterality, hormone receptors, receipt of BCS, Medicaid coverage, year of breast diagnosis and geographic region.

In the unadjusted analysis, there was an increased risk of subsequent MN among breast cancer patients who received RT compared to those who underwent surgery alone (hazard ratio [HR] = 1.38, 95% CI: 1.11–1.72, Table 2). The increased risk of subsequent MN associated with RT receipt persisted in the adjusted analysis (HR = 1.36, 95% CI: 1.03–1.80). After 5 years of follow-up 5.0 (95% CI 2.1–12.2) of 1,000 who received RT and 3.7 (95% CI: 1.8–7.9) of 1,000 who did not receive RT had developed subsequent MN (absolute risk increase of 1.3 per 1,000 patients); corresponding to a number needed to harm of 756 (Fig 2). After 8 years of follow-up, the absolute risk increase was 1.7 per 1,000 patients corresponding to a number needed to harm of 572, consistent with an increase in risk over time and longer follow-up.

**Table 2:** The effect of radiation therapy on subsequent myeloid neoplasm (MN) after breast cancer.

Model Description	Subsequent MN (N = 60,426)	BCS Subgroup, Subsequent MN (N = 38,076)
**Unadjusted Model**	**Hazard Ratio (95% CI)**	**Hazard Ratio (95% CI)**
RT (Claims, ref = No)	**1.38 (1.11–1.72)**	**1.68 (1.20–2.34)**
**Adjusted Model**		
RT (Claims, ref = No)	**1.36 (1.03–1.80)**	**1.51 (1.03–2.22)**
Age (ref = 67–69)		
70–74	0.94 (0.64–1.38)	0.98 (0.62–1.53)
75–79	1.33 (0.93–1.93)	1.19 (0.77–1.85)
80–84	1.11 (0.74–1.66)	1.12 (0.69–1.82)
85+	0.90 (0.53–1.51)	0.79 (0.40–1.58)
Race (ref = White)		
Black	1.29 (0.80–2.06)	159 (0.90–2.81)
Other	1.63 (0.95–2.81)	1.85 (0.98–3.48)
Marital Status (ref = Married)		
Unmarried	0.82 (0.65–1.03)	0.84 (0.63–1.10)
Other	**0.32 (0.12–0.87)**	0.46 (0.17–1.27)
Median household income (ref = <$33,000)		
$33,000–40,000	1.02 (0.67–1.55)	0.97 (0.57–1.66)
$40,000–50,000	1.29 (0.84–1.96)	1.12 (0.66–1.91)
$50,000–63,000	1.00 (0.63–1.59)	0.94 (0.54–1.64)
≥ $63,000	1.14 (0.69–1.90)	0.83 (0.44–1.56)
Education (% adults with High School diploma or less, ref = < 30%)		
30 to < 40%	1.09 (0.76–1.55)	1.22 (0.82–1.84)
40 to <50%	1.20 (0.82–1.75)	1.07 (0.67–1.73)
50 to < 60%	1.05 (0.70–1.59)	1.08 (0.66–1.76)
≥ 60%	0.93 (0.58–1.50)	0.74 (0.40–1.37)
Nonmetropolitan County (ref = Metro)	1.16 (0.83–1.62)	1.05 (0.66–1.65)
Elixhauser Comorbidity (ref = None)		
1 to 2	1.26 (0.97–1.62)	1.26 (0.92–1.72)
3 or more	**1**.**63 (1**.**15–2**.**30)**	**1**.**89 (1**.**25–2**.**86)**
Pre-diagnosis anemia (ref = No)	**2**.**02 (1**.**53–2**.**65)**	**2**.**02 (1**.**45–2**.**82)**
Stage (ref = I)		
In situ	0.91 (0.65–1.28)	0.76 (0.51–1.15)
II	0.92 (0.60–1.42)	1.00 (0.58–1.74)
III	0.78 (0.37–1.64)	1.13 (0.35–3.65)
Grade (ref = Well differentiated)		
Moderately differentiated	1.13 (0.85–1.51)	1.21 (0.86–1.70)
Poorly differentiated	1.18 (0.83–1.66)	1.29 (0.85–1.97)
Undifferentiated	1.48 (0.73–3.00)	1.49 (0.59–3.81)
Unknown	1.13 (0.74–1.72)	0.97 (0.56–1.67)
Tumor size (ref = <2.0 cm)		
2.0-< = 5.0 cm	0.98 (0.63–1.52)	1.03 (0.59–1.82)
>5.0 cm	0.85 (0.30–2.43)	0.83 (0.11–6.30)
Missing	1.05 (0.55–2.00)	1.09 (0.50–2.40)
Lymph node involvement (ref = No positive nodes/Nodes not examined/Missing)	0.90 (0.57–1.43)	1.06 (0.56–1.97)
Left laterality (ref = Right/missing)	1.08 (0.86–1.34)	0.93 (0.71–1.22)
Hormone receptors (ref = ER+ or PR+)		
ER—and PR–	0.80 (0.47–1.39)	0.63 (0.29–1.36)
Missing	0.89 (0.54–1.45)	0.89 (0.47–1.68)
Breast conserving surgery (Claims, ref = Mastectomy)	1.06 (0.78–1.45)	not included
Disability status (ref = Not disabled)	0.86 (0.53–1.40)	0.61 (0.29–1.28)
Medicaid Buy-In (ref = No)	0.56 (0.31–1.02)	0.39 (0.16–0.96)
Year of Diagnosis (ref = 2004)		
2001	1.71 (0.66–4.44)	3.23 (0.84–12.38)
2002	1.49 (0.56–3.94)	2.09 (0.53–8.36)
2003	1.73 (0.67–4.51)	2.85 (0.74–10.98)
2005	0.79 (0.52–1.21)	0.72 (0.42–1.24)
2006	0.74 (0.48–1.15)	0.80 (0.47–1.37)
2007	0.68 (0.43–1.07)	0.76 (0.44–1.31)
2008	0.35 (0.19–0.64)	0.30 (0.14–0.66)
2009	0.45 (0.25–0.79)	0.64 (0.34–1.19)
Geographic Region (ref = Midwest)		
Northeast	0.80 (0.55–1.15)	1.05 (0.66–1.66)
South	0.97 (0.68–1.39)	1.10 (0.67–1.80)
West	0.69 (0.50–0.96)	0.83 (0.54–1.28)

Abbreviations: RT: Radiation Therapy, BCS: Breast Conserving Surgery, MDS: myelodysplastic syndromes, AML: acute myeloid leukemia, ER: estrogen receptor, PR: progesterone receptor, REF: reference.

The positive associations between RT and MN development were consistent when assessed with the SEER-only alternative exposure and outcome measures (adjusted HR = 1.62, 95% CI: 1.10–2.38) (Table 3). Less than half of the cases of MN would have been identified if we had used only SEER to define outcomes, and the difference in cumulative incidence of MN would not have been significant (4.5, 95% CI: 3.5–5.5) in RT-receiving patients versus 4.0 (95% CI: 3.2–5.1) in RT-non-receiving patients per 10,000 person-years, p = .12).

**Table 3:** The effect of radiation therapy on subsequent myeloid neoplasms after breast cancer according to data source (N = 60,426).

		Dependent Variables	
		SEER Only	SEER+Medicare claims
Independent Variables	SEER Only		
	unadjusted	**1.67 (1.19–2.33)**	**1.53 (1.22–1.90)**
	adjusted	**1.62 (1.10–2.38)**	**1.47 (1.12–1.91)**
	SEER+Medicare claims		
	unadjusted	1.31 (0.93–1.83)	**1.38 (1.11–1.72)**
	adjusted	1.16 (0.74–1.81)	**1.36 (1.03–1.80)**

Source: SEER-Medicare. Note: This table shows the effect of RT on development of subsequent myeloid neoplasms according to whether SEER only or SEER+Medicare claims are used to define the variables of interest. The SEER only adjusted model includes the independent variables: radiation therapy (effect shown), age, race, marital status, income, education, metropolitan status, stage, grade, tumor size, node involvement, tumor laterality, hormone receptors, receipt of BCS (SEER defined), Medicaid coverage, year of breast diagnosis and geographic region. The SEER+Medicare adjusted model includes independent variables: radiation therapy (effect shown), age, race, marital status, income, education, metropolitan status, comorbidity, disability, anemia, stage, grade, tumor size, node involvement, tumor laterality, hormone receptors, receipt of BCS (Medicare claims defined), Medicaid coverage, year of breast diagnosis and geographic region.

Finally, in a subset of women who received BCS as their primary surgery, we observed a higher risk of MN associated with RT (adjusted HR = 1.51, 95% CI: 1.03–2.22, Table 2). The adjusted effect of RT on MN did not meet statistical significance in most subgroups defined by disease stage or lymph node involvement (S2 Table).

## Discussion

We found the overall rate of MN among older breast cancer survivors who did not receive chemotherapy to be low. There was a significant increase in risk of subsequent MN diagnosis with RT exposure, though the absolute risk increase was very small. Importantly, we did not observe differences in MN risk associated with type of surgery performed or by stage of breast cancer; characteristics of which could have impacted the extent of RT exposure and potentially have encouraged development of t-MN. Overall, these results should be reassuring for patients and providers deciding about initiating RT after surgery for localized breast cancer.

Indeed, RT after mastectomy has been shown to reduce the absolute risk of 10-year local recurrence by 20% and the absolute 15-year breast cancer mortality by 5.4% in comparison to surgery alone[19]. For women treated with BCS, it has been estimated that RT decreases risk of recurrence by about 50% and the risk of breast cancer-related death by 17%[20]. For counseling and clinical decision making, these benefits of RT are balanced against potential increased risk of second solid and hematologic malignancies. In a large Danish study, among breast cancer with surgery, the risk of RT-associated non-breast solid cancer increased 34% (HR = 1.34, 95% CI: 1.11–1.61) while no increased risk for the non-RT-associated sites (HR = 1.04; 95% CI: 0.94–1.1)[21]. The risk of RT-associated cancer increased over time, and the authors estimated an RT-attributable risk of one second solid cancer for every 200 women treated with RT[21]. In line with these data, after 5 and 8 years of follow-up, we found the number needed to harm to be 756 and 572, respectively, consistent with an increase in cumulative risk over longer follow-up [Fig 2]. In other words, after 8 years of follow-up, 572 women must receive RT for one additional diagnosis of MN to occur. When balanced against the reduction of risks of recurrence and death with RT, our findings suggest that the benefits of RT likely to outweigh the risks of development of MN as well.

Early studies reported increased incidence of t-MN among breast cancer survivors who received RT post-surgery[3]; however, more recent studies questioned whether women who only received RT (without chemotherapy) had an increased incidence of t-MN [S3 Table][4–8, 22–24]. For example, an analysis using the National Comprehensive Cancer Network Breast Cancer Outcomes Database did not find RT alone to be strongly associated with risk of marrow neoplasms (HR 2.6; 95% CI: 0.57 to 11.9; p = .21)[7]. However, the smaller sample size and rarity of events leading to low statistical power might be the reason behind the lack of statistical significance despite the 2.6-fold increase in effect size.

It is well recognized that MN including MDS/AML are underreported to cancer registries including SEER resulting in under-ascertainment of the outcome[25, 26]. If this occurs differentially between those receiving RT and not, it could affect the relative risk calculation. In our analysis, we captured cases of MN in Medicare using a well-defined algorithm that required performance of a bone marrow evaluation[25, 27]. Using this claims-based approach, about half of the captured cases were only recorded in Medicare claims. Similarly, several prior SEER-based analyses used the stage of breast cancer as a proxy for RT and/or chemotherapy use and did not directly ascertain their use since SEER does not reliably capture the use of these modalities[28]. In contrast, we directly ascertained receipt of RT from Medicare claims which is a more reliable measure of RT use. Indeed, relative to our claims-enhanced detailed data, there is potential for measurement error and associated bias in SEER-only analyses.

Our study has other strengths. First, our control group was breast cancer patients who do not receive RT rather than the general population. Patients who develop an initial cancer (e.g. breast cancer) have a higher genetic susceptibility to developing subsequent malignancies. Second, our analysis has a very large sample size (60,436) and a long median follow-up period (5.7 years) with at least three years of potential follow-up for any specific woman. The longer follow-up is especially important as development of t-MN may be associated with a long latency period[29]. Third, many prior analyses reported on either MDS or AML occurrence, but not both. We not only captured AML and MDS, but also included MDS/MPN overlap neoplasms as all these malignancies are considered within the spectrum of t-MN. Finally, newer RT technologies such as intensity-modulated RT (IMRT) and brachytherapy have been proposed to optimize radiation dose delivery and potentially reduce risk of short- and long-term complication, possibly minimizing risk of MDS/AML due to reduced bone marrow exposure[8]. In our analysis, we studied patients who received RT in the modern era who at the same time had a long median follow-up.

We recognize limitations to our work as well. Given the relatively recent addition of Medicare Part D prescription drug claims in the SEER-Medicare data, we lacked adequate sample to assess for potential differential effects of hormonal therapy on occurrence of MN between the RT and non-RT- groups. Due to the relatively small number of diagnosed cases of MN, we were not able to analyze differences between various RT techniques that could be associated with total radiation exposures such as regional versus breast-specific RT delivery or the use of IMRT or brachytherapy. Additionally, it is not feasible using SEER-Medicare data to assess dose or extent of the field irradiated. Moreover, there might be a differential case ascertainment by physicians or patients themselves more aggressively pursuing a diagnosis and work-up for cytopenias among those who have received RT compared those who did not. Additionally, personal risk factors such as smoking history are not available in the dataset. The median follow-up for women who received RT was ten months longer than that of women who did not receive RT (73 vs. 63 months, respectively, p<0.001) which might have led to more cases of t-MN diagnosed in the RT group. Our claims-based algorithm might not have captured all cases of subsequent MN such as those cases in whom the diagnosis was made on peripheral blood findings, however this this lack of capture is not expected to be different between the RT vs no-RT groups. Finally, unobserved confounders may have affected both receipt of RT and patterns of follow-up care leading to identification of t-MN.

In summary, we found that among older breast cancer survivors who did not receive chemotherapy, the overall rate of MN was low. There was a significant increase in the incidence of subsequent MN with RT receipt, but the absolute risk increase was very small. These results are important for both evidence-guided treatment recommendations regarding the use of RT in older patients with breast cancer, and counseling and therapy recommendations for those who develop a MN after RT. Individualized risk assessment should be based on patient and disease risk factors to guide counseling and clinical decision making rather than a general assumption of aggressive MN and poor prognosis merely based on the diagnosis being “therapy-related” just due to occurrence in the post-RT setting.

## Supporting information

S1 Table Administrative codes. Abbreviations: MDS: myelodysplastic syndromes, AML: acute myeloid leukemia, SEER: Surveillance, Epidemiology, and End Results, t-MN: therapy-related myeloid neoplasms, CML: Chronic myeloid leukemia, CMML: Chronic myelomonocytic leukemia, MPN: Myeloproliferative neoplasm.
(DOCX)

S2 Table The effect of RT on subsequent MN after breast cancer according to stage and node involvement. Source: SEER-Medicare. Abbreviations: RT: Radiation Therapy, BCS: Breast Conserving Surgery, MDS: myelodysplastic syndromes, AML: acute myeloid leukemia, ER: estrogen receptor, PR: progesterone receptor, REF: reference.
(DOCX)

S3 Table The effect of RT on subsequent MN after breast cancer according to data source. (DOCX)
