# Supplementary material for: Risk of myeloid neoplasms after radiotherapy among older women with localized breast cancer: A population-based study
Source: PLoS One. 2017 Sep 13;12(9):e0184747. doi: 10.1371/journal.pone.0184747 (PMC5597231; doi:10.1371/journal.pone.0184747)
Supplement: S1 Table — Abbreviations: MDS: myelodysplastic syndromes, AML: acute myeloid leukemia, SEER: Surveillance, Epidemiology, and End Results, t-MN: therapy-related myeloid neoplasms, CML: Chronic myeloid leukemia, CMML: Chronic myelomonocytic leukemia, MPN: Myeloproliferative neoplasm. (DOCX) [file pone.0184747.s001.docx]

**Appendix Table 1. Administrative Codes**

|  | **SEER** | **Claims** |
| --- | --- | --- |
|  | ICD-O-3 Histology | ICD-9 Diagnosis |
| Histology for Breast Sample Inclusion (used in combination with SEER site for breast tumor) | 8000, 8001, 8010, 8020, 8021, 8022, 8050, 8140, 8201, 8211, 8230, 8255, 8260, 8480, 8481, 8490, 8500, 8501, 8502, 8503, 8504, 8507, 8510, 8512, 8513, 8514, 8520, 8521, 8522, 8523, 8524, 8525, 8530, 8540, 8541, 8543, 8570, 8571, 8572, 8573, 8574, 8575 |  |
| MDS | 9980, 9982, 9983, 9985, 9986, 9987, 9989 | 238.7* prior to October 2006, 239.72-239.75 on or after October 2006 |
| AML | 9840, 9861, 9865, 9866 ,9867, 9869, 9870, 9871, 9872, 9873, 9874, 9891, 9893, 9895, 9896, 9897, 9910, 9911, 9920, 9930, 9931, 9984 | 205.0*, 205.3*, 206.0*, 207.0*, 207.2* |
| Other t-MN (including CML, CMML, and MDS/MPN Overlap) | 9920, 9945, 9863, 9875, 9876, 9975 | 205.1* |
| Bone Marrow Biopsy or Aspirate |  | HCPCS 38220, 38221, 85095, 85097, 85102, G0364 or ICD-8-CM procedure 413.1 and 413.8 |

* indicates any code beginning with the specified digits was included. Abbreviations: MDS: myelodysplastic syndromes, AML: acute myeloid leukemia, SEER: Surveillance, Epidemiology, and End Results, t-MN: therapy-related myeloid neoplasms, CML: Chronic myeloid leukemia, CMML: Chronic myelomonocytic leukemia, MPN: Myeloproliferative neoplasm.
