# Supplementary material for: Risk of myeloid neoplasms after radiotherapy among older women with localized breast cancer: A population-based study
Source: PLoS One. 2017 Sep 13;12(9):e0184747. doi: 10.1371/journal.pone.0184747 (PMC5597231; doi:10.1371/journal.pone.0184747)
Supplement: S2 Table — Source: SEER-Medicare. Abbreviations: RT: Radiation Therapy, BCS: Breast Conserving Surgery, MDS: myelodysplastic syndromes, AML: acute myeloid leukemia, ER: estrogen receptor, PR: progesterone receptor, REF: reference. (DOCX) [file pone.0184747.s002.docx]

**Appendix Table 2. The effect of RT on subsequent MN after breast cancer according to stage and node involvement**

Source: SEER-Medicare.

| **Model Description** | **In Situ** | **Stage I** | **Stage II** | **Stage III** | **Node Negative** | **Node Positive** |
| --- | --- | --- | --- | --- | --- | --- |
| **Number in Analysis** | 12,484 | 31,005 | 14,337 | 2,600 | 52,382 | 8,044 |
| **Number (%) who receive RT*** | 5,307 (43%) | 17,008 (55%) | 5,463 (38%) | 981 (38%) | 25,312 (48%) | 3,447 (43%) |
| **Unadjusted Model** |  |  |  |  |  |  |
| RT (Claims, ref*=No) | 0.76 (0.46-1.27) | **1.43 (1.05-1.95)** | **1.78 (1.10-2.87)** | 3.33 (0.84-13.27) | **1.29 (1.02-1.64)** | **2.25 (1.13-4.47)** |
| **Adjusted Model** |  |  |  |  |  |  |
| RT (Claims, ref=No) | 0.87 (0.44-1.72) | **1.53 (1.00-2.34)** | 1.31 (0.76-2.24) | does not converge | **1.41 (1.05-1.91)** | 1.57 (0.67-3.66) |
| Age (ref=67-69) |  |  |  |  |  |  |
| 70-74 | 0.77 (0.37-1.58) | 1.03 (0.62-1.73) | 0.91 (0.31-2.70) |  | 0.96 (0.65-1.44) | 0.70 (0.13-3.75) |
| 75-79 | 1.06 (0.52-2.13) | 1.53 (0.94-2.49) | 1.20 (0.43-3.31) |  | 1.38 (0.95-2.01) | 1.33 (0.28-6.39) |
| 80-84 | 0.58 (0.23-1.41) | 1.12 (0.63-1.98) | 1.46 (0.54-4.00) |  | 1.00 (0.65-1.55) | 2.15 (0.47-9.93) |
| 85+ | 0.74 (0.24-2.26) | 1.29 (0.65-2.57) | 0.48 (0.13-1.76) |  | 0.98 (0.57-1.67) | 0.66 (0.11-4.00) |
| Race (ref=White) |  |  |  |  |  |  |
| Black | 0.79 (0.28-2.17) | **2.20 (1.22-3.98)** | 0.48 (0.15-1.51) |  | 1.30 (0.79-2.14) | 0.67 (0.16-2.89) |
| Other | 0.41 (0.05-3.15) | **1.95 (1.02-3.73)** | 2.32 (0.68-7.92) |  | 1.41 (0.79-2.52) | 3.61 (0.81-16.07) |
| Marital Status (ref=Married) |  |  |  |  |  |  |
| Unmarried | 1.08 (0.65-1.82) | 0.75 (0.55-1.02) | 0.69 (0.42-1.15) |  | 0.83 (0.65-1.06) | 0.66 (0.33-1.32) |
| Other | 0.29 (0.04-2.12) | 0.31 (0.08-1.28) | 0.34 (0.05-2.52) |  | **0.26 (0.08-0.82)** | 0.62 (0.08-4.67) |
| Median household income (ref= <$33,000) |  |  |  |  |  |  |
| $33,000-40,000 | 0.51 (0.18-1.46) | 1.27 (0.70-2.31) | 0.94 (0.46-1.93) |  | 1.02 (0.65-1.62) | 0.99 (0.35-2.81) |
| $40,000-50,000 | 1.30 (0.53-3.21) | 1.64 (0.92-2.91) | 0.67 (0.28-1.62) |  | 1.41 (0.90-2.20) | 0.58 (0.17-2.03) |
| $50,000-63,000 | 0.56 (0.20-1.57) | 1.35 (0.71-2.59) | 0.64 (0.25-1.66) |  | 0.99 (0.60-1.65) | 0.88 (0.26-2.98) |
| ≥ $63,000 | 1.19 (0.41-3.47) | 1.40 (0.71-2.73) | 0.68 (0.20-2.36) |  | 1.22 (0.72-2.09) | 0.62 (0.12-3.17) |
| Education (% adults with High School diploma or less, ref= < 30%) |  |  |  |  |  |  |
| 30 to < 40% | **2.14 (1.04-4.44)** | 0.96 (0.60-1.52) | 0.62 (0.23-1.67) |  | 1.13 (0.79-1.64) | 0.56 (0.16-1.99) |
| 40 to <50 % | 1.74 (0.79-3.81) | 0.86 (0.52-1.43) | 1.71 (0.68-4.32) |  | 1.10 (0.74-1.65) | 1.94 (0.66-5.69) |
| 50 to < 60% | 1.45 (0.61-3.43) | 0.92 (0.54-1.57) | 1.33 (0.48-3.68) |  | 1.05 (0.69-1.60) | 1.05 (0.26-4.34) |
| ≥ 60% | 1.11 (0.38-3.22) | 0.94 (0.51-1.75) | 0.91 (0.30-2.78) |  | 0.93 (0.56-1.55) | 0.76 (0.19-3.10) |
| Nonmetropolitan County (ref=Metro) | 0.98 (0.49-1.97) | **1.70 (1.10-2.62)** | 0.55 (0.25-1.20) |  | 1.27 (0.90-1.80) | 0.42 (0.10-1.67) |
| Elixhauser Comorbidity (ref=None) |  |  |  |  |  |  |
| 1 to 2 | 0.95 (0.52-1.72) | **1.31 (0.93-1.85)** | 1.52 (0.88-2.63) |  | 1.24 (0.94-1.62) | 1.30 (0.61-2.78) |
| 3 or more | 1.89 (0.88-4.05) | **1.86 (1.20-2.89)** | 1.14 (0.53-2.45) |  | 1.70 (1.19-2.43) | 0.96 (0.31-2.95) |
| Pre-diagnosis anemia (ref=No) | 2.31 (1.22-4.37) | **1.58 (1.09-2.29)** | 2.99 (1.67-5.36) |  | 1.96 (1.46-2.62) | 2.34 (1.07-5.13) |
| Grade (ref=Well differentiated) |  |  |  |  |  |  |
| Moderately differentiated | 1.16 (0.43-3.12) | **1.14 (0.81-1.61)** | 1.18 (0.59-2.37) |  | not included | not included |
| Poorly differentiated | 2.37 (0.92-6.14) | **0.91 (0.55-1.51)** | 1.11 (0.51-2.40) |  |  |  |
| Undifferentiated | 1.14 (0.28-4.63) | **2.47 (0.76-8.05)** | 7.14 (2.17-23.41) |  |  |  |
| Unknown | 1.79 (0.69-4.62) | **0.80 (0.41-1.56)** | 1.28 (0.43-3.78) |  |  |  |
| Tumor size (ref= <2.0 cm) |  |  |  |  |  |  |
| 2.0-<=5.0 cm | 1.27 (0.56-2.89) | **1.14 (0.50-2.61)** | 0.49 (0.20-1.20) |  | 0.96 (0.64-1.44) | 0.64 (0.24-1.68) |
| >5.0 cm | no estimate | **not included** | 0.77 (0.15-3.96) |  | 0.63 (0.15-2.57) | 0.74 (0.14-3.90) |
| Missing | 0.85 (0.46-1.57) | **2.17 (0.78-6.04)** | 1.64 (0.50-5.34) |  | 1.31 (0.87-1.98) | 1.73 (0.43-6.94) |
| Lymph node involvement (ref=No positive nodes/Nodes not examined/Missing) | not included | **not included** | 0.64 (0.36-1.15) |  | not included | not included |
| Left laterality (ref=Right/missing) | 0.89 (0.54-1.45) | **1.11 (0.83-1.50)** | 1.24 (0.76-2.02) |  | 1.01 (0.80-1.28) | 1.80 (0.89-3.64) |
| Hormone receptors (ref=ER+* or PR+*) |  |  |  |  |  |  |
| ER– and PR– | 0.26 (0.06-1.07) | **1.34 (0.68-2.65)** | 0.56 (0.13-2.37) |  | 0.81 (0.46-1.44) | 1.44 (0.32-6.57) |
| Missing | 0.91 (0.47-1.76) | **1.06 (0.47-2.42)** | 0.81 (0.31-2.11) |  | 1.13 (0.75-1.71) | 1.43 (0.37-5.54) |
| Breast conserving surgery (Claims, ref=Mastectomy) | 0.71 (0.37-1.35) | **1.00 (0.63-1.58)** | 1.64 (0.95-2.84) |  | 0.94 (0.69-1.28) | 1.64 (0.74-3.68) |
| Disability status (ref=Not disabled) | 0.79 (0.23-2.70) | **0.71 (0.32-1.57)** | 0.94 (0.43-2.08) |  | 0.73 (0.43-1.25) | 1.69 (0.49-5.88) |
| Medicaid Buy-In (ref=No) | 0.87 (0.47-1.61) | **0.67 (0.31-1.45)** | 0.62 (0.24-1.62) |  | 0.88 (0.61-1.26) | 0.31 (0.07-1.31) |
| Geographic Region (ref=Midwest) |  |  |  |  |  |  |
| Northeast | 0.38 (0.19-0.78) | **1.65 (0.94-2.90)** | 0.41 (0.20-0.85) |  | 0.89 (0.60-1.32) | 0.41 (0.17-1.02) |
| South | 0.69 (0.36-1.35) | **1.65 (0.93-2.92)** | 0.60 (0.29-1.25) |  | 1.12 (0.75-1.65) | 0.28 (0.09-0.83) |
| West | 0.38 (0.19-0.76) | **1.42 (0.84-2.40)** | 0.32 (0.17-0.60) |  | 0.82 (0.57-1.17) | 0.19 (0.08-0.46) |

* Abbreviations: RT: Radiation Therapy, ER: estrogen receptor, PR: progesterone receptor, REF: reference.
