# Supplementary material for: Risk of myeloid neoplasms after radiotherapy among older women with localized breast cancer: A population-based study
Source: PLoS One. 2017 Sep 13;12(9):e0184747. doi: 10.1371/journal.pone.0184747 (PMC5597231; doi:10.1371/journal.pone.0184747)
Supplement: S3 Table — (DOCX) [file pone.0184747.s003.docx]

**Appendix Table 3. The effect of RT on subsequent MN after breast cancer according to data source**

| **Study** | **Data source** | **Sample description** | **Treatment/Exposure of interest** | **Outcome** | **Analytic Method** | **Key findings** |
| --- | --- | --- | --- | --- | --- | --- |
| Zeidan A.M. et al (2017)  Current study | SEER-Medicare | N=60,426  In situ to III breast cancer, Age 67-94,  Diagnosed 2001-2009 | Claims defined initial RT vs. no RT (surgery only) within 9 months of diagnosis  Excluded those receiving initial chemotherapy or late RT | Any MN (MDS, AML or Other (CML/CMML/t-MN) | Competing risk Cox model (HR), cumulative incidence rate  Censor follow-up at chemotherapy receipt | RT is associated with a low, but significant risk of subsequent MN among women who had breast cancer |
| Smith R.E. et al (2003) | Six completed National Surgical Adjuvant Breast and Bowel  Project (NSABP) phase III clinical studies | N=8,563  Operable breast cancer, received chemotherapy | Type of breast surgery used as proxy for RT (i.e. lumpectomy patients were considered to have received RT while mastectomy patients were considered not to have received it) | AML and MDS | Stratified log-rank analyses (RR) | Patients who received RT experienced more t-AML/MDS than those who did not (RR=2.38, P=.006) |
| Wolff A.C. et al (2015) | NCCN Breast Cancer Outcomes Database | N=20,063  Stage I to III breast cancer, Age 18-98, Diagnosed 07/1997-12/2007 | Chemotherapy only  vs.  Surgery plus chemotherapy vs. RT + Surgery + chemotherapy | Marrow Neoplasms (including lymphoid N=8 and myeloid neoplasms N=42) | Proportional sub distribution hazards, cumulative incidence rate, Kaplan-Meier model | RT alone not associated with significant increase in MN MDS (HR=2.6; 95% CI: 0.57 to 11.9; P=.21). There is a higher risk of development of marrow neoplasms among patients who received RT post-surgery compared to the general SEER population over the post-diagnosis follow-up period after accounting for age and race effects (observed vs. expected risk, 3.4; 95%CI, 1.4-5.1; p<.001) |
| Kaplan H.G. et al (2004) | One breast cancer registry at a community-based institution and SEER-Medicare | N=2,866  In situ to Stage III breast cancer,  Surgically treated,  Age 21-94, Diagnosed 1992-9,  Mean follow-up of 5.46  Excluded: patients who did not receive surgery, with incomplete chemotherapy records/nonstandard chemotherapy regimens, received stem cell transplantation, lost follow-up or with unknown disease status | No treatment vs. surgery only vs. surgery plus chemotherapy vs. surgery, chemotherapy, and RT | Leukemia, AML, and MDS | Cumulative incidence rate, Kaplan Meier model | No difference between leukemia incidence between patients treated with post-surgical RT and national age-adjusted leukemia incidence among women (0.29% vs. 0.31%) |
| Kaplan H.G. et al (2011) | One breast cancer registry at a community-based institution and SEER 9 Seattle-Puget Sound Registry | N=5,790  In situ to stage III breast cancer,  Surgically treated,  Age 21-94, Diagnosed 1990-2005  Excluded: patients who did not receive surgery, with incomplete chemotherapy records/nonstandard chemotherapy regimens, received stem cell transplantation, lost follow-up or with unknown disease status | No treatment vs. surgery only vs. Surgery plus radiation vs. surgery plus chemotherapy vs. surgery, radiation, and chemotherapy | Leukemia, AML, and MDS | Cumulative incidence rate, Kaplan Meier model | Mean follow-up (7.17 years) for patients diagnosed with breast cancer (stage 0-III) in the 1990-2005 period, the crude AML/MDS incidence was 0.29% (95%CI =0.17,0.47) which was higher than the companion SEER incidence (RR=3.94, 95%CI = 2.34, 6.15), with even high RR (10.9 for MDS and 5.3 for MDS) among patients who were less than 65 years in age at time of breast cancer diagnosis. Among the patients treated with surgery/RT, the RR was also significantly higher at 3.32 (95%CI = 1.42, 6.45) compared to population incidence |
| Kaplan H.G. et al (2013) | SEER | N=306,691  Stage I to III breast cancer, Diagnosed 2001-9 | Stage as a proxy for receipt of RT or chemotherapy | AML and MDS |  | Unadjusted MDS/AML incidence rate was 0.15 % with increasing incidence by age and stage of breast cancer with an overall 2.75 higher risk of being diagnosed with AML/MDS compared to the general population (95%CI, 2.51-3.00) The relative risk for both AML and MDS were again higher for younger women though older breast cancer survivors had also increased risks (those age 65-74 years had RR of 2.94 (95% CI, 2.45, 3.50); and those aged ≥75 years had RR of 1.28 (95%CI, 1.03, 1.56)]. The MDS/AML relative risk increased from 1.87 to 5.66 from stage I-III. |
| Kaplan H.G. et al (2013) | SEER | N=36,191  In situ breast cancer, Any age,  Diagnosed 2001-9 | SEER defined RT | AML and MDS |  | At a mean follow-up of 49.7 months, there was an increased risk for MDS/AML with RT compared to the general population (RR, 2.34, 95%CI, 1.49, 3.46, p <.001) |
| Martin M.G. et al (2009) | SEER | N=420,076  Stage I and III breast cancer, Any age, Diagnosed 1973-2002 | SEER defined RT | AML |  | There is an age-dependent risk of a subsequent diagnosis of AML in women diagnosed with breast cancer who were less than 50 years in age (RR 4.14; P <.001) and those aged 50-64 years (RR 2.19; P <.001), but not among women who were 65 years or older (RR 1.19; P =.12) when compared with the expected incidence of AML in the population. Similar to Kaplan and colleague, the authors noticed a stage-dependent increase in RR of AML among younger patients with stage III breast cancer when compared with those who had stage I disease at diagnosis (RR, 2.92; P=.004), but not in older women (RR=0.79; P=.80), which could partially be explained by greater chemotherapy exposure. |
| Yu, G. Et al (2006) | SEER | Three diagnosis based cohorts: 1975-7, 1983-5, and 1991-3 |  | Any second cancer |  | In 8 years after initial breast cancer diagnosis. Using the 1990s data, the authors noted that RT slightly increased the risks of second leukemia (RR=1.8, 95% CI, 1.2-2.8) |
| Chen Y. et al (2015) | Medical records from University of Texas M.D. Anderson Cancer Center | N=235 women diagnosed with t-MN or second MN in 1983-2009 and with a history of breast cancer, 453 women with de novo MN | N/A | AML and MDS | Kaplan Meier model, Cox proportional hazards model (HR) | Therapy-related disease is not an independent risk factor in patients with myeloid neoplasms and with a history of breast cancer. This study used age-matched general population as a control for RT-receiving breast cancer patients rather than using breast cancer patients who do not receive RT as the control group. This is an important issue since it is well documented that patients who develop a cancer (e.g. breast cancer) have a higher genetic susceptibility to develop subsequent malignancies, compared with the general population regardless of exposure to any cytotoxic therapies and therefore choosing the general population as a control group can confound assessment of risk associated with RT. |

References^1-9^

1. Chen Y, Estrov Z, Pierce S, et al. Myeloid neoplasms after breast cancer: "therapy-related" not an independent poor prognostic factor. *Leuk Lymphoma* 2015; **56**(4): 1012-9.

2. Kaplan H, Malmgren J, De Roos AJ. Risk of myelodysplastic syndrome and acute myeloid leukemia post radiation treatment for breast cancer: a population-based study. *Breast Cancer Res Treat* 2013; **137**(3): 863-7.

3. Kaplan HG, Malmgren JA, Atwood M. Leukemia incidence following primary breast carcinoma treatment. *Cancer* 2004; **101**(7): 1529-36.

4. Kaplan HG, Malmgren JA, Atwood MK. Increased incidence of myelodysplastic syndrome and acute myeloid leukemia following breast cancer treatment with radiation alone or combined with chemotherapy: a registry cohort analysis 1990-2005. *BMC cancer* 2011; **11**: 260.

5. Kaplan HG, Malmgren JA, Li CI, Calip GS. Age related risk of myelodysplastic syndrome and acute myeloid leukemia among breast cancer survivors. *Breast Cancer Res Treat* 2013; **142**(3): 629-36.

6. Martin MG, Welch JS, Luo J, Ellis MJ, Graubert TA, Walter MJ. Therapy related acute myeloid leukemia in breast cancer survivors, a population-based study. *Breast Cancer Res Treat* 2009; **118**(3): 593-8.

7. Smith RE, Bryant J, DeCillis A, Anderson S, National Surgical Adjuvant B, Bowel Project E. Acute myeloid leukemia and myelodysplastic syndrome after doxorubicin-cyclophosphamide adjuvant therapy for operable breast cancer: the National Surgical Adjuvant Breast and Bowel Project Experience. *J Clin Oncol* 2003; **21**(7): 1195-204.

8. Wolff AC, Blackford AL, Visvanathan K, et al. Risk of marrow neoplasms after adjuvant breast cancer therapy: the national comprehensive cancer network experience. *J Clin Oncol* 2015; **33**(4): 340-8.

9. Yu GP, Schantz SP, Neugut AI, Zhang ZF. Incidences and trends of second cancers in female breast cancer patients: a fixed inception cohort-based analysis (United States). *Cancer Causes Control* 2006; **17**(4): 411-20.
